# Microneedle Array Technique for the Longitudinal Extraction of Interstitial Fluid without Hair Removal

**DOI:** 10.3390/mps5030046

**Published:** 2022-06-03

**Authors:** Robert M. Taylor, Abdul-Mehdi S. Ali, Yiliang Zhu, Alicia M. Bolt, Justin T. Baca

**Affiliations:** 1 Department of Emergency Medicine, The University of New Mexico, Albuquerque, NM 87131, USA; rmtaylor@salud.unm.edu; 2 Department of Earth and Planetary Sciences, The University of New Mexico, Albuquerque, NM 87131, USA; mehdiali@unm.edu; 3 Department of Internal Medicine, The University of New Mexico, Albuquerque, NM 87131, USA; yizhu@salud.unm.edu; 4 Department of Pharmaceutical Sciences, College of Pharmacy, The University of New Mexico, Albuquerque, NM 87131, USA; ambolt@salud.unm.edu

**Keywords:** microneedle, interstitial fluid, heavy metals, inductively coupled plasma mass spectrometry

## Abstract

Interstitial fluid (ISF) bathes the cells and tissues and is in constant exchange with blood. As an exchange medium for waste, nutrients, exosomes, and signaling molecules, ISF is recognized as a plentiful source of biomolecules. Many basic and pre-clinical small animal studies could benefit from an inexpensive and efficient technique that allows for the in vivo extraction of ISF for the subsequent quantification of molecules in the interstitial space. We have previously reported on a minimally invasive technique for the extraction of ISF using a 3D-printed microneedle array (MA) platform for comprehensive biomedical applications. Previously, hairless animal models were utilized, and euthanasia was performed immediately following the procedure. Here, we demonstrate the technique in Sprague Dawley rats, without the need for hair removal, over multiple extractions and weeks. As an example of this technique, we report simultaneous quantification of the heavy metals Copper (Cu), Lead (Pb), Lithium (Li), and Nickel (Ni) within the ISF, compared with whole blood. These results demonstrate the MA technique applicability to a broader range of species and studies and the reuse of animals, leading to a reduction in number of animals needed to successfully complete ISF extraction experiments.

## 1. Introduction

Pre-clinical development of novel diagnostics and therapies requires not only a concrete understanding of circulating biomarkers, but also an understanding of how circulating biomarkers compare with tissue-level expression. Serum, plasma, and urine are commonly utilized biofluids; however, interstitial fluid (ISF) is also recognized as a plentiful source of biomolecules [1,2,3,4,5]. ISF bathes the cells and tissues, is in constant exchange with blood, and acts as an exchange medium for waste, nutrients, exosomes, and signaling molecules [5,6]. Biomolecules quantified in ISF often have comparable levels to those found in serum, plasma, and whole blood [2,6,7], suggesting that ISF sampling could replace blood collection. However, unique biomolecules have also been identified in ISF compared to serum and plasma [2,3], which could result in novel biomarker identification.

Sampling ISF for basic and pre-clinical animal studies shows promise, with numerous examples of ISF monitoring of specific molecules [8,9,10,11,12]. However, there remains a need for an inexpensive and facile technique that extracts ISF in vivo for general analysis. A simple ISF extraction technique could supplement or replace blood collection in a variety of time course studies. Techniques for sampling ISF have evolved rapidly [2,6,13]. We previously reported on a minimally-invasive technique for the extraction of ISF using a 3D-printed microneedle array (MA) [1,2,3,14]. While this technique enables a variety of ISF analysis approaches [1,2,3], hairless animal models have been used, and euthanasia was performed following the procedure. Here, we demonstrate the technique in a widely used rat model, namely Sprague Dawley rats, without the need for hair removal, over multiple extractions and weeks. As an example of this technique, we report the simultaneous quantification of the heavy metals Copper (Cu), Lead (Pb), Lithium (Li), and Nickel (Ni) within the ISF, compared with whole blood. These developments allow for the use of a broader range of species and studies and the reuse of animals, leading to a reduction in the number of animals needed to complete ISF extraction experiments. 

As an example, chronic exposure to heavy metals (HMs) is associated with many detrimental health effects, including cardiovascular disease, cancer, reproductive problems, kidney disease, and liver damage [15,16,17,18,19]. HM contamination in soil and water costs trillions of dollars annually to the U.S. and global economies in remediation and health costs [20,21]. As an example, Ni is a heavy metal that has been implicated in numerous medical conditions, including cancer, lung fibrosis, contact dermatitis, asthma, and cardiovascular disease [15,16]. Jewelry is commonly made from Ni, and prolonged contact with the skin can lead to Ni ions being absorbed through the skin. This leads to allergic effects in some individuals. To date, the authors are only aware of one study that examined the heavy metal concentrations in ISF. Bonde et al. [22] used a suction-blister microneedle technique to extract ISF from 12 women with a nickel allergy (ISF successfully extracted from 10 subjects; 83% success rate), compared with individuals with no known Ni allergy. Atomic Absorption Spectroscopy (AAS) was then used to quantify the Ni in the ISF. Their results suggest that the Ni concentration in the individuals with nickel allergy were significantly lower than the controls. The authors suggested that an interesting question, warranting further study, is whether the Ni differences are due to possible differences in cellular uptake. However, the authors also suggested that the suction blister microneedle technique of ISF extraction may also lead to escape of serum components through the microvasculature. Additionally, the suction blister technique inherently relies on localized trauma, in the form of a blister, which likely causes inflammation, separation of the dermal layers, and molecular changes within the ISF [2,23,24]. To demonstrate the applicability of our minimally invasive MA technique to longitudinal studies in haired animals, we simultaneously quantified the baseline Cu, Li, Ni, and Pb concentrations in the MA-extracted ISF of Sprague Dawley rats with ad lib access to tab water over 8 weeks, using inductively coupled plasma-Mass spectrometry (ICP-MS). 

## 2. Materials and Methods

**Figure 1:**
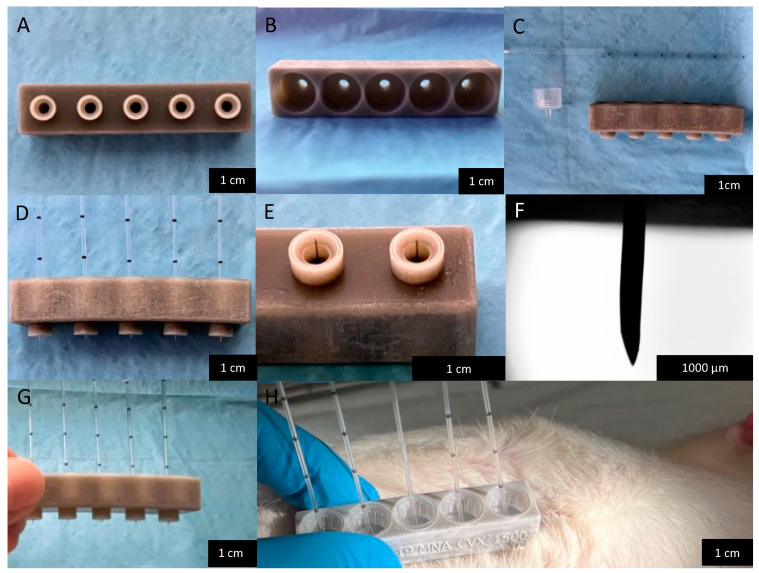
Microneedle Array (MA) is shown from the bottom side (**A**) and top side (**B**). All three pieces of the assembly (needle, capillary tube, and microneedle array) are shown in (**C**). The fully assembled MA is shown in (**D**), and a closer image of the MA tip with needles protruding ~1500 µm is shown in (**E**). Light microscopy was used to visualize the needle tip length protruding from the tip of the MA in (**F**). The fully assembled MA being held in a hand is shown as a size reference in (**G**). Application of the MA in a Sprague Dawley rat with no hair removal is shown in (**H**).

The animal care and use program of The University of New Mexico (UNM) is accredited by AAALAC International, and it approved all experiments (#19-200827-HSC). A total of three, 7–10-week-old, CD^®^ hairless, Crl:CD-Prss8hr, rats (2 female, 1 male) (Charles River Laboratories, Wilmington, MA, USA) and six, 5–6-week-old, Sprague Dawley, Crl:SD, rats (3 female, 3 male) (Charles River Laboratories, Wilmington, MA, USA) were used. Animals were anesthetized with 2.0 % Isoflurane using a nose cone. The Sprague Dawley rats were used for longitudinal studies to determine the baseline Cu, Pb, Li, and Ni concentrations in ISF, compared with whole blood. Formal power calculations to prespecify sample size were not possible due to the preliminary nature of this study. Ultra-fine Nano PEN needles (BD, Franklin Lakes, NJ, USA) were placed into MA holders [1,14] (Figure 1) and attached to calibrated pipet capillary tubes (Drummond Scientific Co., Broomall, PA, USA). The array assembly [1,14] was pressed onto the abdominal dermal tissue of the rats until a sufficient volume of ISF was collected. ISF, collected from the six Sprague Dawley animals in the longitudinal study, was transferred into microcentrifuge tubes containing 20 µL of HPLC-grade nitric acid for Inductively Coupled Plasma-Mass Spectrometry (ICP-MS). The six animals in the longitudinal study were removed from anesthesia following the microneedle applications. Rats were monitored during recovery, returned to their cage, and allowed to recover for 6 days under daily monitoring.

On day 7, the above MA procedure was repeated. This process of ISF extraction, 6-day recovery, and ISF Extraction was repeated for 8 weeks. All animals had a terminal cardiac puncture under heavy anesthesia at the conclusion of each experiment. 

Samples were transferred into digestion tubes (15 mL), and the sample containers were rinsed with 200 µL of Ultra High Purity (UHP)-grade nitric acid (HNO_3_). Samples were then digested at 95 °C using a small digestion block for about 15 min. Samples were then cooled and brought to a final volume of 10 mL with 18-mega Ohm water to match the matrix of the calibration standards. The samples tubes were then transferred into SeaFast or PrepFast autosampler racks for analysis using the PerkinElmer NexION300D ICP/MS.

The ICP/MS was optimized using a tuning solution for a wide range of masses. Both systems (SeaFast and ICP/MS) were conditioned twice using 2% HNO_3_ (UHP Grade). The ICP/MS was calibrated using a blank and four calibration standards, ranging from 1.25–10.0 µg/L. Two calibration verification quality control samples (Initial Calibration Blank Verification “ICBV” and Initial Calibration Verification “ICV”) were analyzed after calibration standards to verify their accuracy. Samples were analyzed, and a Continuing Calibration Verification (CCV) quality control sample was analyzed at a frequency after every 20 samples to validate instrument and calibration stability. Data were verified, validated, exported, and reported via an Excel file. The data analysis was performed using Excel and Python. 

## 3. Results

**Figure 2:**
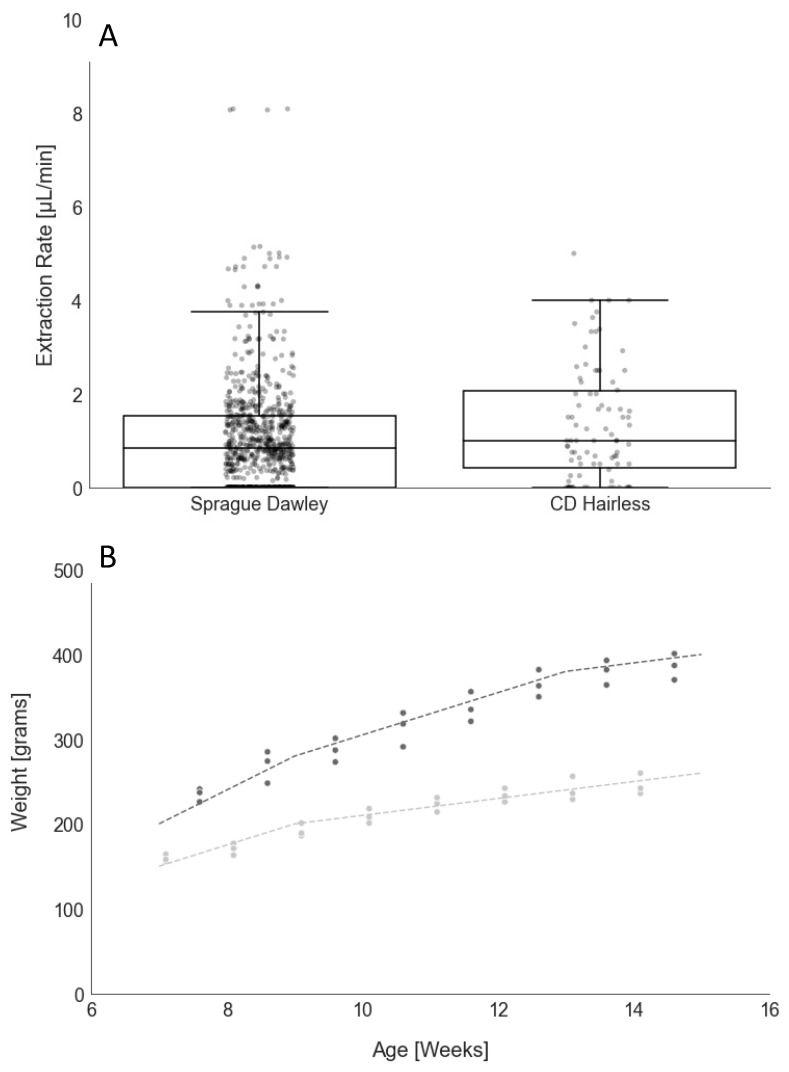
ISF Extractions in CD^®^ Hairless and Sprague Dawley Rats. (**A**) Extraction rate (µL/min) is shown for each individual microneedle array application for the CD^®^ Hairless (N = 3, *n* = 41) and Sprague Dawley (N = 6, *n* = 208) rats on the dot plots. N = number of animals, and *n* = number of MA applications. An underlay boxplot is shown representing the extraction rate quartiles. (**B**) Growth rates for the female (light gray) and male (dark gray) Sprague Dawley rats included in the longitudinal study. Dashed lines show representative data extrapolated from growth charts from the supplier [25].

We successfully extracted ISF, using our MAs (Figure 1), from all CD^®^ Hairless and unshaven Sprague Dawley rats, with average ISF extraction rates per application of 1.26 ± 1.00 µL/min and 0.81 ± 0.83 µL/min, respectively (Figure 2A). As expected, extraction rates were higher for the hairless animals; however, extraction rates were sufficient for the collection of up to 10 µL of ISF in under 30 min from the unshaven Sprague Dawley rats in the longitudinal study.

There were no significant changes in mean fluid volume or extraction rates in the Sprague Dawley animals from week to week over the eight-week longitudinal study. A single application is defined as one MA insertion. Multiple MA insertions were performed per animal. No special preparation, such as shaving, was used prior to extraction. Table 1 shows characteristics of these extractions. ISF was collected in 89.5% and 63.9% of MA applications in CD^®^ Hairless and Sprague Dawley rats, respectively, and we were successful in collecting ISF from 100% of all hairless and unshaven haired rats. Additionally, no adverse events, such as lethargy or changes in appetite, water consumption, or physical appearance, were observed for any of the 6 animals in the longitudinal study. No weight loss was evident, and all 6 animals had growth curves consistent with the supplier (Figure 2B). 

**Figure 3:**
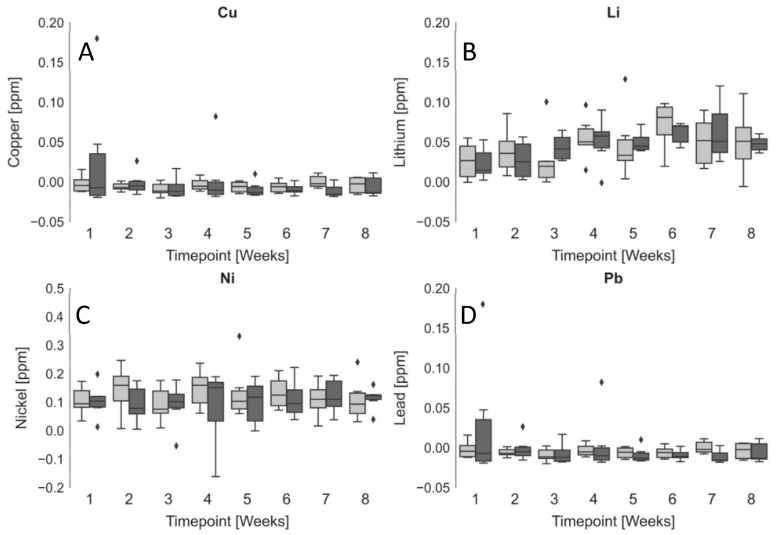
Heavy Metals in Unexposed Sprague Dawley Rat ISF and Blood over 8 weeks. ICP-MS quantification of (**A**) Copper, (**B**) Lithium, (**C**) Nickel, and (**D**) Lead in ISF (light grey) and whole blood (dark grey). Number of animals (N) = 6 (3 female and 3 male). Boxplots show the median (solid line), minimum (bottom whisker), lower quartile (bottom of box), upper quartile (top of box), maximum (upper whisker), outliers (diamonds) ♦, and interquartile range (gray area).

The six Sprague Dawley animals (unexposed) had ad lib access to tap water without any additional heavy metals added. As reference, the Environmental Protection Agency (EPA) sets the maximum contaminant level (MCL) of Cu, Li, Ni, and Pb in drinking water at 1.3 ppm, 0 ppm, 0.2 ppm, and 0.006 ppm, respectively [26,27]. ISF was extracted every 7 days for 8 weeks. At the time of each extraction, blood was also collected through a tail snip. The ISF and whole blood samples had all four heavy metals simultaneously quantified using ICP-MS. Figure 3 shows the unexposed blood vs. ISF concentrations for Cu, Pb, Li, and Ni. We found no significant difference between the ISF and blood concentrations of Cu (0.005 ± 0.034 and –0.001 ± 0.025 ppm, respectively), Li (0.046 ± 0.034 and 0.046 ± 0.025 ppm, respectively), Ni (0.122 ± 0.069 and 0.111 ± 0.108 ppm, respectively), or Pb (0.005 ± 0.070 and −0.001 ± 0.032 ppm, respectively). There were no significant changes in heavy metal concentrations from week one through to eight in either ISF or blood. The similarity between the ISF and blood concentrations of each of the heavy metals suggests that MA-extracted ISF may be a useful surrogate for blood in clinical applications and could be useful as a fluid for minimally invasive remote monitoring of chronic heavy metal exposures in the field. To our knowledge, this is the first report on the simultaneous quantification of multiple heavy metals in ISF in vivo. 

## 4. Discussion

This expansion of our MA ISF technique into unshaven haired animals is safe over multiple repeated procedures and over multiple weeks. This allows for the re-use of animals and a reduction in the number of animals needed for ISF extraction experiments. This development has also demonstrated the applicability in haired animals without the need for shaving. Eliminating the need for shaving reduces the time under anesthesia, total experiment time, and animal stress. This also opens the door for a much broader spectrum of ISF studies in possibly a much broader range of haired species. All previous publications on ISF extractions have described hairless or shaved animal models and/or alternate extraction techniques, such as the suction-blister technique. These techniques require either the formation of a blister, shaving, negative pressure, or the application of depilatory agents [23,24,28]. Each of these can have unwanted consequences to the concentrations of biomolecules in the ISF due to tissue injury and inflammation. We have established an MA design with greater spacing between microneedles and potential space in between contact sites, which allows for similar extraction rates in both hairless and haired animals, without the need for hair removal (1.26 ± 1.00 µL/min and 0.81 ± 0.83 µL/min, respectively). Although variability was evident in the ISF extraction rates, this variability was very similar between the two strains of rats. Additionally, individual animal and device factors may contribute to this variability. Skin thickness was not investigated in this study, as it requires biopsy or necropsy. We have previously investigated the anatomical positioning of the extractions, as well as different tip designs of the MA [14]. Future experiments to further define what variables contribute to the extraction of ISF will increase the applicability of this method. 

Additionally, we found that MA-extracted ISF may be a useful surrogate for blood for minimally invasive remote monitoring of chronic heavy metal exposures in the field. We measured Copper, Lead, Lithium, and Nickel in the ISF and blood of six Sprague Dawley rats and found that concentrations in ISF and blood were not significantly different: Cu (0.005 ± 0.034 and −0.001 ± 0.025 ppm), Li (0.046 ± 0.034 and 0.046 ± 0.025 ppm), Ni (0.122 ± 0.069 and 0.111 ± 0.108 ppm), or Pb (0.005 ± 0.070 and −0.001 ± 0.032 ppm), respectively. Future studies investigating ISF concentrations of other heavy metals such as arsenic, cadmium, and uranium may shed more light on the distribution of heavy metals within the ISF, their toxicity, and methods for remotely monitoring subjects with chronic heavy metal exposures, such as those living near abandoned uranium mines. 

## Figures and Tables

**Table 1:** Characteristics of ISF extractions.

Strain	# MA Insertions	Extraction Rate [µL/min]	STDEV	% Animals with ≥6.5 µL ISF Extracted	% MA Applications with ≥0.5 µL ISF Extracted
**CD^®^ Hairless**	41	1.26	1.00	100 (*n* = 3)	89.5
**Sprague Dawley**	208	0.81	0.83	100 (*n* = 6)	64.0

## Data Availability

The data presented in this study are available on request from the corresponding author.
